# Analysis of Rare Variants in Genes Related to Lipid Metabolism in Patients with Familial Hypercholesterolemia in Western Siberia (Russia)

**DOI:** 10.3390/jpm11111232

**Published:** 2021-11-19

**Authors:** Elena Shakhtshneider, Dinara Ivanoshchuk, Olga Timoshchenko, Pavel Orlov, Sergey Semaev, Emil Valeev, Andrew Goonko, Nataliya Ladygina, Mikhail Voevoda

**Affiliations:** 1 Institute of Cytology and Genetics (ICG), Siberian Branch of Russian Academy of Sciences (SB RAS), 10 Prospekt Ak. Lavrentyeva, 630090 Novosibirsk, Russia; dinara@bionet.nsc.ru (D.I.); lentis@yandex.ru (O.T.); orlovpavel86@gmail.com (P.O.); sse85@ngs.ru (S.S.); emil@bionet.nsc.ru (E.V.); mvoevoda@ya.ru (M.V.); 2 Institute of Internal and Preventive Medicine (IIPM)—Branch of ICG SB RAS, 175/1 Borisa Bogatkova Str., 630089 Novosibirsk, Russia; 3 Department of Automation, Novosibirsk State Technical University, 20 Prospekt K. Marksa, 630073 Novosibirsk, Russia; gun@ait.cs.nstu.ru (A.G.); natskanzler@gmail.com (N.L.)

**Keywords:** familial hypercholesterolemia, targeted sequencing technologies, multiplex ligation-dependent probe amplification, *LDLR*, *APOB*, *ABCG5*, *APOC3*, *LPL*, *SREBF1*, rare variants

## Abstract

The aim of this work was to identify genetic variants potentially involved in familial hypercholesterolemia in 43 genes associated with lipid metabolism disorders. Targeted high-throughput sequencing of lipid metabolism genes was performed (80 subjects with a familial-hypercholesterolemia phenotype). For patients without functionally significant substitutions in the above genes, multiplex ligation-dependent probe amplification was conducted to determine bigger mutations (deletions and/or duplications) in the *LDLR* promoter and exons. A clinically significant variant in some gene associated with familial hypercholesterolemia was identified in 47.5% of the subjects. Clinically significant variants in the *LDLR* gene were identified in 19 probands (73.1% of all variants identified in probands); in three probands (11.5%), pathogenic variants were found in the *APOB* gene; and in four probands (15.4%), rare, clinically significant variants were identified in genes *LPL*, *SREBF1*, *APOC3*, and *ABCG5*. In 12 (85.7%) of 14 children of the probands, clinically significant variants were detectable in genes associated with familial hypercholesterolemia. The use of clinical criteria, targeted sequencing, and multiplex ligation-dependent probe amplification makes it possible to identify carriers of rare clinically significant variants in a wide range of lipid metabolism genes and to investigate their influence on phenotypic manifestations of familial hypercholesterolemia.

## 1. Introduction

Familial hypercholesterolemia is a condition caused by a type of genetic defect leading to a decreased rate of removal of low-density lipoproteins from the bloodstream and a pronounced increase in the blood level of total cholesterol [1]. Familial hypercholesterolemia is one of the most common congenital metabolic disorders [2]. With very rare exceptions, familial hypercholesterolemia is an autosomal dominant disorder [3]. In patients with familial hypercholesterolemia, there is a significantly higher total serum cholesterol level (from birth) and a significantly higher low-density lipoprotein cholesterol (LDL-C) concentration, while triglyceride levels are normal or moderately elevated [4]. Familial hypercholesterolemia poses a substantial risk of the early onset of complications such as coronary heart disease and atherosclerotic lesions in the vessels of the brain and arteries of the lower extremities [5]. In people with a high blood level of LDL-C and a confirmed mutation associated with familial hypercholesterolemia, the incidence of cardiovascular diseases is 22-fold higher than that in people with a normal level of LDL-C and no mutation [6]. Despite the considerable prevalence of this disease and the availability of effective treatments, familial hypercholesterolemia often remains undiagnosed and untreated, especially in children [7].

*LDLR*, *APOB*, and *PCSK9* are the genes whose mutations underlie the pathogenesis of the autosomal dominant type of familial hypercholesterolemia. Mutations in the *LDLR* gene are present in 80–85% of cases in which a molecular genetic cause of familial hypercholesterolemia has been identified; *APOB* mutations are present in 5–7% of the patients; and *PCSK9* mutations have been identified in fewer than 5% of the patients. Mutations in genes associated with the autosomal recessive type of familial hypercholesterolemia—for example, in the *LDLRAP1* gene—are seen in <1% of the cases [8,9]. A negative result of genetic screening for mutations of *LDLR*, *APOB*, *PCSK9*, *LDLRAP1*, and some other genes does not rule out familial hypercholesterolemia. In 5–30% of its cases, molecular genetic testing does not show changes in the above-mentioned genes: some cases of phenotypic familial hypercholesterolemia may be caused by mutations in other genes (currently not known to be associated with this disease) or may have a polygenic cause, in contrast to the autosomal dominant type of this disease [10,11,12,13,14].

More than 250 loci associated with dyslipidemia are known thanks to exome sequencing [15,16,17]. Targeted sequencing of a set of genes of interest is a fast and cost-effective alternative to exome sequencing and is widely used in clinical laboratories. The aim of the present work was to identify genetic variants potentially involved in familial hypercholesterolemia in 43 genes associated with lipid metabolism disorders; for this purpose, we analyzed patients having a phenotype of familial hypercholesterolemia.

## 2. Materials and Methods

### 2.1. Patients

Patients with familial hypercholesterolemia were recruited in the Clinical Diagnostic Department of the Institute of Internal and Preventive Medicine (IIPM)—a branch of the Institute of Cytology and Genetics, Siberian Branch of Russian Academy of Sciences (ICG SB RAS). The study protocol was approved by the Ethics Committee of the IIPM—a branch of the ICG SB RAS (session No. 68 of 4 June 2019). Written informed consent to be examined and to participate in the study was obtained from each patient or his/her parent or legal guardian.

The study included 80 patients referred by a clinical lipidologist for molecular genetic testing after a diagnosis of familial hypercholesterolemia. This diagnosis was made using the Dutch Lipid Clinic Network (DLCN) Criteria [Geneva: World Health Organization; 1999]. To calculate the total score in accordance with the DLCN criteria, the Familial Hypercholesterolemia Calculator software was used, which has been developed in the Department of Automation of the Faculty of Automation and Computer Engineering at Novosibirsk State Technical University (https://lipidcenter.iimed.ru/o-lipidnom-tsentre/calc.html [accessed on 1 November 2021]). In this software, the likelihood of familial hypercholesterolemia in a patient is computed from the sum of subscores obtained in each group of criteria. For these calculations, LDL-C levels of first-degree relatives that are known to exceed the age- and sex-specific 95th percentile are analyzed by the software according to populational age- and sex-specific LDL-C data from Russia [1]. Familial hypercholesterolemia is designated as “definite,” “probable,” or “possible” according to the total score (hereafter: DLCN score). All three subtypes are referred to as a “phenotype of familial hypercholesterolemia” below. A JavaScript v.1.5 function is employed to compute the total score. Taking into account the DLCN criteria and the results of molecular genetic testing, an electronic database was compiled for this study.

In this way, in the total of 80 participants, 34 subjects were assigned to the “definite” familial hypercholesterolemia group (DLCN score > 8), five subjects to the “probable” familial hypercholesterolemia group (DLCN score: 6–8), and 41 subjects to the “possible” familial hypercholesterolemia group (DLCN score: 3–5). The study included 60 probands and 20 first-degree relatives of the probands. Among these relatives, 70% (*n* = 14) were children of the probands.

The patients underwent a medical examination and ultrasonographic testing, and blood samples were collected for biochemical tests (the lipid profile and general biochemical indicators) and molecular genetic testing. The testing of the probands and their first-degree relatives was carried out according to the principles of cascade genetic screening [18].

Blood samples for biochemical tests were taken once from the cubital vein in the morning on an empty stomach (12 h after a meal). The lipid parameters (levels of total cholesterol, triglycerides, LDL-C, and high-density lipoprotein cholesterol [HDL-C]) and blood glucose concentration were determined by enzymatic methods on an automatic biochemical analyzer KoneLab300i (Vantaa, Finland) with Termo Fisher reagents (Vantaa, Finland). The LDL-C level was calculated using the Friedewald formula; when LDL-C concentration was >4.5 mmol/L, direct LDL-C measurement was employed. Means ± standard deviation were computed in the SPSS software for Windows for the data on biochemical testing of the subjects over 18 years of age.

### 2.2. Isolation of Genomic DNA

To isolate DNA from blood, phenol–chloroform extraction was used [19]. The quality of the extracted DNA was assessed by means of an Agilent 2100 Bioanalyzer capillary electrophoresis system (Agilent Technologies Inc., Santa Clara, CA, USA).

### 2.3. Genome Library Preparation, Sequencing, and Bioinformatic Analysis

Targeted high-throughput sequencing of lipid metabolism genes was performed on the whole participants (*n* = 80, all had a diagnosis of familial hypercholesterolemia to various degrees of certainty). The custom panel of genes for the testing consisted of 43 genes (*LDLR*, *APOB*, *PCSK9*, *LDLRAP1*, *CETP*, *LPL*, *HMGCR*, *NPC1L1*, *PPARA*, *MTTP*, *LMF1*, *SAR1B*, *ABCA1*, *ABCG5*, *ABCG8*, *CYP7A1*, *STAP1*, *LIPA*, *PNPLA5*, *APOA1*, *APOA5*, *APOC2*, *APOE*, *LCAT*, *ANGPTL3*, *LIPC*, *APOA4*, *APOC3*, *SREBF1*, *LMNA*, *PPARG*, *PLIN1*, *POLD1*, *LPA*, *SMAD1*, *SMAD2*, *SMAD3*, *SMAD4*, *SMAD5*, *SMAD6*, *SMAD7*, *SMAD9*, and *LIPG*). Genes *LDLR*, *APOB*, and *PCSK9* are associated with the autosomal dominant type of familial hypercholesterolemia; genes *LDLRAP1*, *ABCG5*, *ABCG8*, and *CYP7A1* with the autosomal recessive type of familial hypercholesterolemia; and genes *STAP1*, *LIPA*, and *PNPLA5* are associated with phenotypic variations of familial hypercholesterolemia, including the development of hypertriglyceridemia. Rare variants in the genes of apolipoproteins and other genes related to lipid metabolism can cause a familial-hypercholesterolemia-like phenotype in patients [20,21,22,23,24].

Targeted high-throughput sequencing (next-generation sequencing) was performed with NimbleGen SeqCap Target Enrichment (Roche, Basel, Switzerland) on a MySeq sequencer (Illumina, San Diego, CA, USA). The coverage was 97%.

The bioinformatic processing pipeline included the following steps. After we removed adapters from sequencing data via cutadapt (available online: https://cutadapt.readthedocs.io/en/stable/ [accessed on 4 October 2021]), sequencing reads were mapped with a Burrow–Wheeler Alignment tool (BWA) v.0.7.17 (available online: http://bio-bwa.sourceforge.net/ [accessed on 4 October 2021]); we used human genome assembly GRCh38 as a reference. At the second stage, sequencing data were improved: we removed PCR-generated duplicates with MarkDuplicates by Picard (available online: https://broadinstitute.github.io/picard/ [accessed on 4 October 2021]), and recalibrated base quality with BQSR tools of Genome Analysis Toolkit (GATK) v.3.3 (available online: https://gatk.broadinstitute.org/hc/en-us [accessed on 4 October 2021]). At the third stage, we performed single-nucleotide variant (SNV) calling via HaplotypeCaller tool of GATK.

Finally, SNVs were annotated and filtered. Annotation was performed by means of the ANNO-VAR tool (available online: https://annovar.openbioinformatics.org/en/latest/ [accessed on 4 October 2021]); we utilized the following databases:

− Big population frequency databases, such as gnomAD (available online: https://gnomad.broadinstitute.org/ [accessed on 4 October 2021]), with some help of databases on specific populations, like Greater Middle East (GME) Variome Project (available online: http://igm.ucsd.edu/gme/ [accessed on 4 October 2021]), AbraOM (Brazilian genomic variants) (available online: https://abraom.ib.usp.br/ [accessed on 4 October 2021]), and Korean Personal Genome Project (available online: http://opengenome.net/Main_Page [accessed on 4 October 2021]).
− Databases representing in silico prediction tools, such as dbNSFP (available online: https://sites.google.com/site/jpopgen/dbNSFP [accessed on 4 October 2021]), which contains data from more than 30 pathogenicity prediction tools (e.g., MutationTaster2, SIFT, PROVEAN, and Polyphen2), and from 10 conservation prediction tools (e.g., phastCons, GERP++, and SiPhy). For pathogenicity prediction tools, we set thresholds according to respective authors’ recommendations; additionally, for conservation prediction tools, we used one common threshold, 0.7; therefore, we assumed a variant to be conserved if its conservation score was greater than the scores of ≥70% other variants. Additionally, we used databases dbscSNV and regSNP-intron for variants that may have an effect on splicing. Nevertheless, all these databases were only a supplementary tool, and they contributed little to the summary measure of pathogenicity.
− Gene-based phenotype databases (e.g., OMIM).
− Clinical significance databases: ClinVar (available online: https://www.ncbi.nlm.nih.gov/clinvar/ [accessed on 4 October 2021]), Human Gene Mutation Database (HGMD) (available online: http://www.hgmd.cf.ac.uk/ [accessed on 4 October 2021]), and Leiden Open Variation Database (available online: https://www.lovd.nl/ [accessed on 4 October 2021]); variants described as benign (B) or likely benign (LB) were also excluded from further analysis.
− PubMed (available online: https://pubmed.ncbi.nlm.nih.gov/ [accessed on 4 October 2021]) and some other article databases as a source of information on specific clinical cases.

We estimated pathogenicity of each novel candidate mutation according to the recommendations of the American College of Medical Genetics and Genomics (ACMG) and the Association for Molecular Pathology [25].

### 2.4. Verification of Findings

All selected rare SNVs were verified by Sanger direct automatic sequencing on an ABI 3500 DNA sequencer (Thermo Fisher Scientific, Waltham, MA, USA) by means of the BigDye Terminator v3.1 Cycle Sequencing Kit (Thermo Fisher Scientific, Waltham, MA, USA). Primer design for the selected SNVs was performed in the Primer-Blast software (available online: https://www.ncbi.nlm.nih.gov/tools/primer-blast/ [accessed on 4 October 2021]). Targeted Sanger sequencing was carried out to detect mutations in the relatives of the probands.

### 2.5. Multiplex Ligation-Dependent Probe Amplification (MLPA)

For patients without functionally significant point substitutions in the above genes, MLPA was conducted to find possible bigger mutations (deletions and/or duplications) in the *LDLR* promoter and exons. The MLPA analysis was performed using SALSA MLPA Kit P062 (MRCHolland, Amsterdam, The Netherlands) followed by the separation of fluorescently labeled fragments by capillary electrophoresis (on an ABI3500 sequencer). The Coffalyser.Net software (MRCHolland, Amsterdam) was utilized to interpret the MLPA results.

## 3. Results

In the subjects over 18 years of age (*n* = 69; all participants [*n* = 80] had a phenotype of familial hypercholesterolemia), the total cholesterol level was 8.6 ± 3.4 mmol/L (mean ± standard deviation), with a maximum of 25 mmol/L (Table 1). This relatively low total cholesterol concentration is due to the fact that at the time of the initial medical examination by the clinical lipidologist in the Clinical Diagnostic Department of the IIPM (a branch of the ICG SB RAS), lipid-lowering drugs were taken by 41% of the probands. All lipid-lowering drugs were statins. One proband did not tolerate lipid-lowering medication of the statin class. According to the medical examination, in 42% of the subjects, tendon xanthomas were present. Three probands (4.9%) had comorbid type 2 diabetes mellitus.

A clinically significant variant in some genes associated with familial hypercholesterolemia was identified in 47.5% of the subjects. Clinically significant variants in the *LDLR* gene were identified in 19 probands (73.1% of all variants identified in probands); in three probands (11.5%), pathogenic variants were found in the *APOB* gene, and in four probands (15.4%), rare, clinically significant variants were identified in genes *LPL*, *SREBF1*, *APOC3*, and *ABCG5*. In 12 (85.7%) of 14 children of the probands, clinically significant variants were detectable in genes associated with familial hypercholesterolemia: in 10 cases, in the *LDLR* gene; in one case, in the APOB gene; and in one case, in the *SREBPF1* gene. Among the other six first-degree relatives of the probands (mother, father, or sibling), a pathogenic variant in the *LDLR* gene was identified in one case.

### 3.1. LDLR

Structural changes in the *LDLR* gene in patients with a phenotype of familial hypercholesterolemia are presented in Table 2. All missense variants were heterozygous. Some of the identified variants (Cys352Tyr, Cys340Phe, and Leu401His) have been described in patients with familial hypercholesterolemia in Russia [26,27,28,29,30]. The variant most common in our participations—rs121908038—was found in three unrelated families (six subjects total).

Two unrelated probands were compound heterozygotes in terms of the *LDLR* gene and showed a clinical course corresponding to the homozygous type of the disease. In the first case, a 28-year-old (patient P52) got a diagnosis of “definite” familial hypercholesterolemia (DLCN score of 18, a history of myocardial infarction at the age of 24; percutaneous transluminal coronary angioplasty at ages 25 and 26 years); rare variants chr19:11217342 and chr19:11221441 were identified in exons 5 and 7 of the *LDLR* gene (Table 2). The patient had been taking lipid-lowering medication, and the total cholesterol level was 18.6 mmol/L, and LDL-C 15.2 mmol/L.

In the second case, a 35-year-old (patient P10) got a diagnosis of “definite” familial hypercholesterolemia (DLCN score of 18, a history of myocardial infarction at the age of 24; mammary coronary artery bypass grafting at the age of 24; a second myocardial infarction at 34 years of age); rare variants were identified in exons 3 and 8 of the *LDLR* gene. One of these variants is located at position chr19:11213415 (NM_000527:exon3:c.G266C:p.C89S), earlier, a rare “pathogenic” variant (rs875989894) has been described at this position: a G > A substitution (available online: https://databases.lovd.nl/shared/variants/0000092111#00011039 [accessed on 1 October 2021]). The other variant is located at chr19:11222252; it is described for the first time: NM_000527:exon8:c.T1123G:p.Y375D (Table 2). Before treatment with lipid-lowering medication was started, the total cholesterol level was 25.0 mmol/L, and LDL-C 11.94 mmol/L.

The clinical signs of the homozygous type of familial hypercholesterolemia, including early onset of severe complications in the cardiovascular system, are typical for patients who are compound heterozygotes in the *LDLR* gene, as shown in various populations [34].

**Figure 1:**
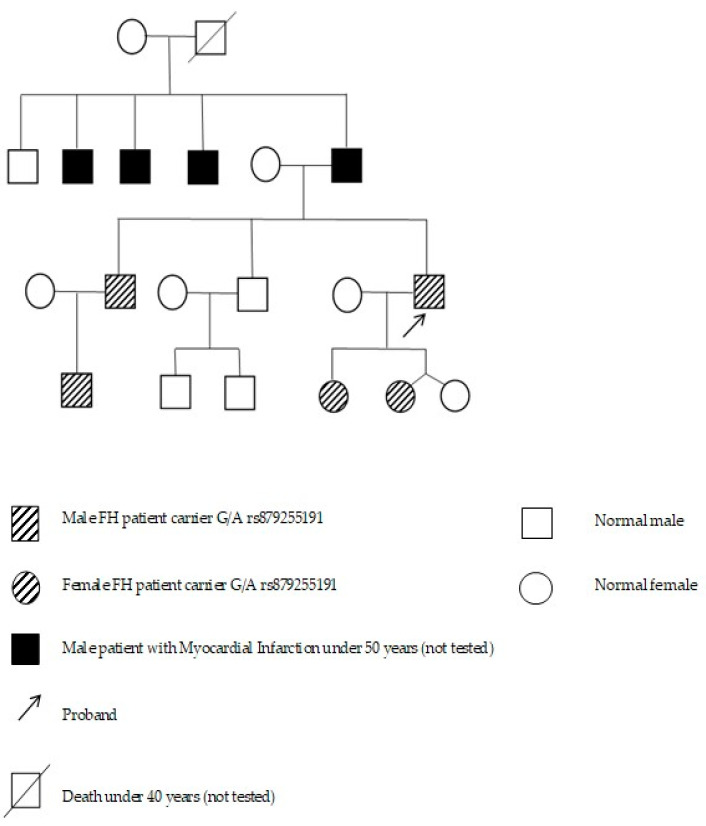
The screened family with variant rs879255191 identified in the *LDLR* gene.

The rs879255191 variant in the regulatory region of *LDLR* has previously been described by us in proband P24 with familial hypercholesterolemia and in his two children (patients P25 and P26) with hyperlipidemia (7 and 8 years old) [35,36]. Variant c.2389 + 5G > A is located in highly conserved dinucleotide AG at the splice donor site of intron 16 of *LDLR*. Functional significance of the detected substitution was evaluated in the SPANR software (available online: http://tools.genes.toronto.edu/ [accessed on 4 October 2021]). According to the SPANR analysis, the change in the probability of exon inclusion in mRNA in various tissues when this variant is present is −13.47 points, meaning that the probability of inclusion of exons 14–16 in gene transcripts is lower than 100%. Previously, rs879255191 has already been detected in patients with familial hypercholesterolemia, including in Russia [32]. In the present study, the rs879255191 variant was also detected in a sibling (patient P81) and a nephew (P82) of the proband (P24) who has clinical signs of familial hypercholesterolemia (Figure 1). Patient P81 had a myocardial infarction at age 44 years.

### 3.2. Identification of Deletions and Duplications in the LDLR Gene by MLPA

**Figure 2:**
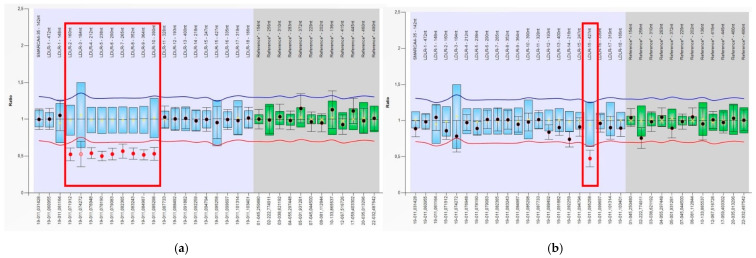
MLPA analysis shows a heterozygous deletion (ratio of ~1.5) of *LDLR* exons 2–10 in patient P33 (**a**) and of *LDLR* exon 15 in patient P5 (**b**). The ratio charts of the patients with a familial hypercholesterolemia phenotype were generated by means of SALSA MLPA Kit P062. Each red dot below the red curve represents a deletion. The probes whose data do not go outside the blue and red curves represent two wild-type copies of the *LDLR* gene.

Forty-two patients, without functionally significant point substitutions in lipid metabolism genes, were subjected to MLPA analysis to find possible structural changes (deletions or duplications) in the *LDLR* promoter and exons. This analysis revealed deletions in DNA samples from two unrelated patients. In the first case, deletion NM_000527.4:c.(67+1_68-1)_(1586+1_1587-1)del in a heterozygous state eliminated a region spanning exons 2 to 10 (Figure 2a). In the second case, the patient was a carrier of a deletion of exon 15 in the *LDLR* gene NM_000527.4:c.(2140+1_2141-1)_(2311+1_2312-1)del in a heterozygous state (Figure 2b).

Patient P33 (31 years old) with an exon 2–10 deletion had a total cholesterol level of 10.96 mmol/L, and LDL-C 7.76 mmol/L (DLCN score: 6), with a family history of deaths from myocardial infarction before the age of 50 on the paternal side. At the time of the medical examination, she was not taking lipid-lowering medication. This deletion was first described in 2010 in the French Autosomal Dominant Hypercholesterolemia Research Network study [37].

In patient P5 (69 years old) with an exon 15 deletion, the total cholesterol level was 6.6 mmol/L and LDL-C 3.8 mmol/L while the patient was on 10 mg of rosuvastatin. A similar deletion of *LDLR* exon 15 has been described by Koivisto P.V. et al. [38].

### 3.3. APOB

In the molecular genetic test, we detected some structural changes in the *APOB* gene in patients with a phenotype of familial hypercholesterolemia (Table 2). In three patients from two unrelated families (a proband and a son of the proband from one family and a proband from another family), variant rs5742904 was found: NM_000384.3(APOB):c.10580G > A (p.Arg3527Gln). Minor allele frequency was T = 0.000275 according to database GnomAD. 

In the first family, patient P71 (36 years old) carried the rs5742904 variant, and the total cholesterol level was 7.75 mmol/L and LDL-C 5.76 mmol/L while on 10 mg of rosuvastatin (DLCN score: 12; examination revealed xanthomas). The patient’s father had a myocardial infarction at age 50 and died of a second myocardial infarction at age 62; the father’s mother died of ischemic stroke at age 60. The son (13 years old, patient P11) of patient P71—had a total cholesterol level of 6.9 mmol/L and carried the rs5742904 variant.

In the second family, patient P15 (25 years old) carried the rs5742904 variant, and the total cholesterol level was 7.5 mmol/L, LDL-C 5.7 mmol/L, and the DLCN score was 11.

The clinical effect of rs5742904 has conflicting interpretations of pathogenicity in the literature: likely pathogenic (three studies), pathogenic (27 studies), and uncertain significance (one study) according to database ClinVar [39].

### 3.4. ABCG5

A rare variant, rs145164937, in the *ABCG5* gene in a heterozygous state was detected in one of the probands (patient P74, woman, 38 years old) with a total cholesterol level of 8.3 mmol/L, LDL-C of 5.4 mmol/L, and a DLCN score of 3 (Table 2). Minor allele frequency (MAF) of allele C is 0.002223 for Europeans (non-Finnish) according to the GnomAD database [40]. A known disease-associated mutation at this position is HGMD ID CM169023. When the rs145164937 (NM_022436; p.Ala98Gly; c.293C > G) variant was analyzed in PolyPhen-2, it showed a high probability of a damaging effect on the protein product (a score of 0.977). Earlier, an association of rs145164937 with non-high-density lipoprotein cholesterol levels has been demonstrated [41].

### 3.5. APOC3

Analysis of the targeted-high-throughput-sequencing data from the *APOC3* gene revealed variant rs138326449 (IVS2 + 1G > A) in a heterozygous state (Table 2). MAF of allele C is 0.002244 for Europeans (non-Finnish) according to the GnomAD database. This substitution is located in a highly conserved dinucleotide at a splice donor site, thereby leading to alternative splicing and a dysfunctional protein [42]. A.B. Jorgensen et al. have reported an association of the IVS2 + 1G > A variant with low triglyceride levels and a low risk of coronary heart disease [42]. In our study, variant IVS2 + 1G > A of *APOC3* was detected in a 65-year-old subject (patient P59) with hypercholesterolemia (total cholesterol 8.2 mmol/L, LDL-C 5.45 mmol/L) and normal levels of triglycerides (0.7 mmol/L) and HDL-C (2.4 mmol/L), with a DLCN score of 5. In the ClinVar database, rs138326449 is listed as pathogenic for apolipoprotein C-III deficiency.

### 3.6. LPL

Analysis of the targeted-high-throughput-sequencing results from the *LPL* gene revealed a rare variant: rs118204077 (NM_000237; p.Arg270Cys, c.808C > T) in a heterozygous state (Table 2). MAF of allele C is 0.0001 for Europeans (non-Finnish) according to the GnomAD database. Its clinical significance is indicated as pathogenic in the ClinVar database. A known disease-associated mutation at this position is HGMD ID CM941054. When the rs118204077 variant was analyzed in PolyPhen-2, the probability of a damaging effect on the protein product turned out to be 1.000 (very high score).

The rs118204077 variant in *LPL* was identified in a 45-year-old (patient P9) with hypercholesterolemia (12.4 mmol/L), hypertriglyceridemia (17.4 mmol/L), and a DLCN score of 5. Previously, at this locus, variants associated with hypertriglyceridemia have been described in the literature [43,44].

### 3.7. SREBF1

A rare variant of the *SREBF1* gene (rs115855236, chr17:17820281) was identified in proband P83 and a son of this proband (patient P84) with clinical signs of familial hypercholesterolemia (Table 2). MAF of allele A is 0.001210 for Europeans (non-Finnish) according to the GnomAD database. In other genes associated with familial hypercholesterolemia, pathogenic and probably pathogenic variants were not identified in these patients. *SREBF1* encodes a transcription factor of the basic helix-loop-helix-leucine zipper family (bHLH-Zip); this protein binds to a sterol regulatory element (SRE1) and regulates the biosynthesis of fatty acids and cholesterol. One of the target genes for transcription factor SREBF1 is *LDLR*.

## 4. Discussion

In this study, the prevalence of detected “pathogenic” and “probably pathogenic” mutations was 47.5% among the analyzed probands with a phenotype of familial hypercholesterolemia from the Western Siberia region (Russia) and 85.7% among their tested children who also had a phenotype of familial hypercholesterolemia. As in other studies, this finding confirms the effectiveness of cascade genetic screening [45,46,47,48,49,50].

Among 34 subjects with a diagnosis of “definite” familial hypercholesterolemia (DLCN score > 8), pathogenic variants in *LDLR* (76.5%) or *APOB* (8.8%) were identified in 29 subjects. In five subjects (14.7%), no pathogenic variants were found in the genes included in the tested panel.

Among the five subjects with a diagnosis of “probable” familial hypercholesterolemia (DLCN scores 6–8), two subjects were found to have pathogenic variants in the *LDLR* gene, including deletion NM_000527.4:c.(67+1_68-1)_(1586+1_1587-1)del in a heterozygous state.

Among 41 subjects with a diagnosis of “possible” familial hypercholesterolemia (DLCN score: 3–5), rare “pathogenic” and “probably pathogenic” variants in the analyzed genes of lipid metabolism were identified in only 15% of cases. In two subjects, pathogenic variants in the *LDLR* gene were identified, including a deletion of the 15th exon NM_000527.4:c.(2140+1_2141-1)_(2311+1_2312-1)del; in one subject, a pathogenic variant was found in the *APOB* gene, and in four probands, in other lipid metabolism genes (*ABCG5*, *LPL*, *APOC3*, or *SREBF1*). A negative result of genetic screening for *LDLR*, *APOB*, and *PCSK9* mutations does not rule out the presence of familial hypercholesterolemia in a patient. In ~40% of cases, molecular genetic testing fails to determine the cause of familial hypercholesterolemia [51] or an elevated LDL-C level that may have a polygenic type of inheritance [52,53]. Despite the lower prevalence of detected mutations among our subjects with “possible” familial hypercholesterolemia, they should also be monitored by a doctor and receive appropriate lipid-lowering therapy because of the high risk of cardiovascular events at elevated LDL-C levels and/or with a family history of an early cardiovascular disease [54,55,56].

Molecular genetic research on familial hypercholesterolemia in Russia has been conducted for more than 30 years in different regions of the country [29]. It is worth mentioning some variants of the *LDLR* gene that not only occur in most regions of Russia but are also the most common variants of this gene: rs121908038 and rs761954844 [28,29,30,31,32,57]. Additionally, these variants have been found in populations of Northern and Central Europe (rs121908038) and in populations of Central and Eastern Europe, Southeast Asia, and North America (rs761954844) [40].

The most common limitation of such studies is a small sample size [29]. We should also mention low accessibility of molecular genetic testing in some regions of Russia and the high cost of these tests. The small sample sizes do not allow us to assess clinical-course features of the disease that are associated with various pathogenic variants in lipid metabolism genes and to evaluate the spectrum of pathogenic variants in lipid metabolism genes in the population of Russia. Targeted sequencing can be useful not only for rapid and cost-effective diagnosis of familial hypercholesterolemia, but also for investigation of rare variants of lipid metabolism genes and their influence on the patients’ phenotype [30,57,58]. This method may help to combine the efforts of physicians and investigators from different regions for the research on familial hypercholesterolemia. 

MLPA is necessary for the diagnosis of familial hypercholesterolemia in patients without functionally significant point substitutions in relevant genes to which one of the sequencing methods has been applied [59]. Wider use of the MLPA method should help to identify 2–3% more probands with familial hypercholesterolemia. As revealed by research on the practice of molecular genetic diagnostics, patients who carry a confirmed pathogenic variant in a relevant gene are more likely to lead a healthy lifestyle and to regularly take lipid-lowering medication [18].

The use of clinical criteria, targeted sequencing, and MLPA makes it possible to identify carriers of rare clinically significant variants in a wide range of lipid metabolism genes and to investigate their influence on phenotypic manifestations of familial hypercholesterolemia.

## Figures and Tables

**Table 1:** Data on biochemical testing of the subjects over 18 years of age, *n* = 69.

	M ± SD *	Minimum	Maximum
Glucose, mmol/L	5.7 ± 1.2	4.0	10.4
Total cholesterol, mmol/L (mg/dL)	8.6 ± 3.4 (332.5 ± 131.4)	3.4 (131.4)	25.0 (966.5)
Triglycerides, mmol/L (mg/dL)	1.8 ± 1.4 (157.5 ± 122.5)	0.4 (35.0)	17.4 (1522.5)
HDL-C, mmol/L (mg/dL)	1.4 ± 0.4 (54.1 ± 15.5)	0.54 (20.9)	2.2 (85.1)
LDL-C, mmol/L (mg/dL)	5.6 ± 2.3 (216.5 ± 88.9)	1.11 (42.9)	11.94 (461.6)
Body–mass index, m^2^/kg	27.2 ± 4.6	19.0	39.0
Age, years	46.0 ± 13.9	20	73

* M ± SD: mean ± standard deviation.

**Table 2:** The genetic variants identified in Western Siberia patients with a phenotype of Familial Hypercholesterolemia. MAF: minor allele frequency.

Patient ID	dbSNP ID	Position on Chromosome (GRCh38)	Nucleotide Substitution	Amino Acid Substitution	MAF According to Database GnomAD	Clinical Effect According to Database ClinVar	References for Russia
*LDLR* Gene							
P28, P40, P41, P42, P55, P56	rs121908038	19:11113293	c.1202T > A	p.Leu401His	ND	Likely Pathogenic	Zakharova et al., 2005 [26]; Shakhtshneider et al., 2017 [27]; Shakhtshneider et al., 2019 [28]; Vasilyev et al., 2020 [29]; Miroshnikova et al., 2021 [30]
P45	rs137853964	19:11129602	c.2479G > A	p.Val827Ile	A = 0.001006	Likely Pathogenic	Shakhtshneider et al., 2017 [27]; Shakhtshneider et al., 2019 [28]; Vasilyev et al., 2020 [29]
Р22, P36, P58	rs28942078	19:11113376	c.1285G > A	p.Val429Met	A = 0.000012	Pathogenic/Likely Pathogenic	-
P65	rs539080792	19:11221396	c.1009G > A	p.Glu337Lys	A = 0.000104	Uncertain Significance	Shakhtshneider et al., 2017 [27]; Shakhtshneider et al., 2019 [28]; Vasilyev et al., 2020 [29]
P47	rs570942190	19:11113337	c.1246C > T	p.Arg416Trp	T = 0.000024	Not reported in ClinVar	Shakhtshneider et al., 2017 [27]; Shakhtshneider et al., 2019 [28]; Vasilyev et al., 2020 [29]
P67, P68	rs755757866	19:11110730	c.1019G > T	p.Cys340Tyr	T = 0.000008	Likely Pathogenic	Shakhtshneider et al., 2017 [27]; Shakhtshneider et al., 2019 [28]; Vasilyev et al., 2020 [29]
Р7	rs761954844	19:11110697	c.986G > A	p.Cys329Tyr	A = 0.000016	Likely Pathogenic	Zakharova et al., 2005 [26]; Shakhtshneider et al., 2019 [28]; Semenova et al., 2020 [31]; Vasilyev et al., 2020 [29]; Miroshnikova et al., 2021 [30]
P35	rs879254566	19:11105440	c.534TT > G	p.Asp178Glu	ND	Pathogenic/ Likely Pathogenic	Shakhtshneider et al., 2019 [28]; Vasilyev et al., 2020 [29]
P38, P39	rs879254721	19:11107496	c.922G > A	p.Glu308Lys	ND	Pathogenic	Semenova et al., 2020 [31]; Vasilyev et al., 2020 [30];
P2	rs879254980	19:11116179	c.1672G > T	p.Glu558Ter	ND	Pathogenic	-
P24, P25, P26, P81, P82	rs879255191	19:11128090	c.2389 + 5G > A	-	ND	Conflicting Interpretations of Pathogenicity	Meshkov et. Al. [32]; Shakhtshneider et al., 2019 [28]; Vasilyev et al., 2020 [29]
P52	rs875989907	19:11106666	c.796G > A	p.Asp266Asn	A = 0.000012	Pathogenic	Shakhtshneider et al., 2017 [27]
	rs879254769	19:11110765	c.1054T > C	p.Cys352Ser	ND	Likely Pathogenic	Shakhtshneider et al., 2017 [27]
P10	rs875989894	19:11213415	c.266G > C	p.Cys89Ser	ND	Pathogenic/Likely Pathogenic	-
	ND	19:11222252	c.1123T > G	p.Tyr375Asp	ND	Novel variant	-
*APOB* Gene							
P11, P15, P71	rs5742904	2:21006288	c.10580G > A	p.Arg3527Gln	T = 0.000275	Pathogenic	Voevoda et al. 2014 [33]; Shakhtshneider et al., 2019 [28]; Miroshnikova et al., 2021 [30]
*ABCG5* Gene							
P74	rs145164937	2:43832056	c.293C > G	p.Ala98Gly	C = 0.002223	Conflicting interpretations of pathogenicity	-
*APOC3* Gene							
P59	rs138326449	11:116830638	c.55 + 1G > A	-	C = 0.002244	Conflicting interpretations of pathogenicity	-
*LPL* Gene							
P9	rs118204077	8:19955873	c.808C > T	p.Arg270Cys	C = 0.0001	Pathogenic	-
*SREBF1* Gene							
P83, P84	rs115855236	17:17820281	c.422C > T	p.Pro141Leu	A = 0.001210	Not reported in ClinVar	-

## Data Availability

The data presented in this study are available on request from the corresponding author. The data are not publicly available due to privacy concerns.
